# Patient perspectives on group problem management plus for adults with major depressive disorder in rural Malawi

**DOI:** 10.1080/16549716.2025.2500785

**Published:** 2025-05-09

**Authors:** Owen Mwale, Karen Kasambara, Amruta Houde, Kondwani Mpinga, Waste Kayira, Michael Harawa, Myrrah Kamwiyo, Rachel Isaacs, Basimenye Nhlema, Todd Ruderman, Olive Liwimbi, Michael Udedi, Ksakrad Kelly, Ryan K. McBain

**Affiliations:** a APZU, Partners In Health, Neno, Malawi; b Ministry of Health, Zomba Mental Hospital, Zomba, Malawi; c Clinical Services, Ministry of Health, Lilongwe, Malawi; d Headquarters, Partners In Health, Boston, MA, USA; e Healthcare Delivery, RAND Corporation, Washington, DC, USA

**Keywords:** Depression, mental health, care integration, task shifting, psychotherapy, key informant interviews

## Abstract

**Background:**

Major depressive disorder (MDD) frequently co-occurs with other medical conditions. Care integration and task shifting are two frameworks that may strengthen person-centered care among individuals with MDD and comorbid diagnoses, including for adults with limited access to healthcare resources living in rural settings within sub-Saharan Africa.

**Objective:**

We assessed the acceptability and feasibility of group psychotherapy (Problem Management Plus [PM+]) integrated into chronic healthcare services in Neno District, based on key informant (KI) interviews with clients who received PM+ services from local counselors.

**Methods:**

We conducted in-depth interviews with 31 KIs, comprising adult patients participating in group PM+ in rural Malawi. The interview covered facets such as knowledge acquisition, logistical considerations for organizing PM+ sessions, selection of appropriate venues, session format, and overall perceived acceptability. Themes were identified through thematic content analysis.

**Results:**

We identified five emergent themes: limited prior awareness and understanding of MDD, positive elements of the PM+ service delivery model, patients’ perceived effectiveness of PM+, logistical challenges with effectively engaging PM+, and positive views on acceptability of PM+. Findings revealed a strong appreciation and enthusiasm for PM+, although KIs noted areas for improvement – including lengthy travel times to receive PM+, limited compensation and privacy, and counselors arriving late.

**Conclusion:**

Insights from clients underscore the potential utility of group PM+ as a task-shifted model of MDD care that can be integrated into existing service packages in resource-limited settings, as well as opportunities for improvements such as reducing travel time to care and identifying venues with greater client privacy.

**Trial Registration:**

ClinicalTrials.gov identifier: NCT04777006.

## PAPER CONTEXT

- **Main findings:** Adult clients with major depressive disorder who received group Problem Management Plus, a form of psychotherapy, perceived the intervention and delivery model to be effective and acceptable, but they noted several areas for improvement.
- **Added knowledge:** The findings of this study demonstrate the acceptability and feasibility of Problem Management Plus for wider scale adoption among adults with major depressive disorder in Malawi.
- **Global health impact for policy and action:** This study contributes to a growing corpus of evidence about the potential utility of the public sector adopting task-shifted mental health interventions to address widespread mental health needs in resource-limited settings.

## Background

Major depressive disorder (MDD) is one of the leading contributors to disability worldwide [1]. In Malawi, the prevalence of MDD ranges from 3% to 18%, depending on the population and setting [2–4]. Higher rates are observed among individuals with chronic illnesses, HIV/AIDS, and those experiencing socioeconomic hardships [2–4].

MDD frequently co-occurs with other medical conditions such as diabetes, HIV, and hypertension [5,6], and these conditions share a bidirectional relationship with MDD in which each can exacerbate the other [7]. For instance, individuals with HIV face a higher risk of developing MDD. Additionally, MDD can emerge as a side effect of certain medication treatments [8]. Similarly, insulin resistance or type 2 diabetes can increase the likelihood of depression or worsen existing depressive symptoms [9]. Evidence shows that these comorbidities can negatively impact treatment adherence and symptom remission among those with MDD, especially in low-resource settings where a large majority of individuals with MDD fail to receive treatment in the first place [10].

To address these challenges, care integration and task-shifting strategies have been employed. While care integration supports patient-centered care by addressing the needs of patients in a more holistic manner, task-shifting transfers care delivery from a limited number of highly trained personnel to non-specialized healthcare providers within the local setting [11]. Evidence from low-resource settings indicates that task-shifted models of mental health services can be delivered through brief interventions in community-based settings to bolster sustainability and financial feasibility of scale-up, and numerous studies have found positive impacts on population health outcomes [12,13]. More recently, structured frameworks and guidelines for implementation of task-shifting, along with deployment policies, have been developed to bolster success in sub-Saharan African contexts [14–16].

Over the past several years, researchers have demonstrated that Problem Management Plus (PM+) is an effective psychological intervention suitable for addressing common mental health conditions such as depression and anxiety [12]. PM+ is a brief psychological intervention aimed at alleviating symptoms of depression, anxiety, and distress by incorporating cognitive behavioral therapy (CBT) and problem-solving therapy (PST) principles [17]. Studies have demonstrated that PM+, in both its individual and group formats, is effective and can be adapted for delivery by trained helpers, including non-specialists in community-based settings using a manualized protocol [18–20]. The brief intervention – consisting of five multi-hour sessions – is designed for low- and middle-income countries (LMICs) where limited capacity often poses implementation challenges for staffing, supervision, physical space, and time commitments [21].

While implementation challenges pose a threat to the feasibility of PM+, acceptability is an additional area of concern. In many LMICs, conditions such as MDD are stigmatized: they are often viewed as the result of drug abuse or demon possession [22]. Additionally, individuals may be unaware of or skeptical about psychiatric conditions [23], or else skeptical of treatments that do not involve a pharmacological component. Lastly, MDD often limits individuals’ energy and motivation, and in rural areas the burden of travel to and from health care services may lead individuals to cease coming [24].

The implementation of PM+ in patients with comorbid chronic medical conditions such as HIV, diabetes, and hypertension, alongside MDD, has not been explored. Moreover, only a few studies have explored the acceptability of group PM+ (rather than one-on-one PM+), a framework recently developed by WHO that can generate cost-savings by treating multiple patients simultaneously [19]. This study aims to provide insights on the application of group PM+ in this population, with a specific emphasis on perceived feasibility and acceptability of group PM+ from the patient perspective – by conducting 30 key informant interviews. The study is couched within a broader clinical trial in a rural district of Malawi, which aims to assess the effectiveness of a stepped approach to MDD treatment that comprises group PM+ and antidepressant therapy [4]. We hypothesized that, as a result of adaptations to the local setting, group PM+ would be deemed acceptable and feasible for broader implementation, but that participants would identify ongoing barriers and opportunities to strengthen the model of care delivery.

## Methods

### Study design

This qualitative study was part of a cluster randomized controlled trial for integrating MDD treatment into chronic care clinics throughout Neno District, Malawi [25]. We focused on understanding experiences and perceptions of group PM+ as the primary modality of MDD treatment – from the perspectives of patients who participated in one or more group PM+ sessions. Key informant interviews, rather than focus group discussions, were selected because of the logistical challenges presented in convening participants in a setting as large as Rhode Island, mountainous, and lacking transportation infrastructure.

### Study setting

Integrated Chronic Care Clinics in Neno District, Malawi were established in 2014 with the goal of introducing screening and treatment for non-communicable diseases into Malawi’s HIV service platform. This initiative specifically targets patients with chronic medical conditions, including HIV, diabetes, asthma, epilepsy, hypertension, and psychosis – across all 14 health facilities throughout Neno District [26,27].

For the clinical trial, MDD screening and treatment were incorporated into Neno District’s Integrated Chronic Care Clinic (IC3) paradigm. Patients attending IC3 underwent depression screening using the PHQ-9 questionnaire [28], a validated screening tool for assessing depressive symptoms in Malawi. Patients with moderate or severe symptoms (PHQ-9 scores ≥ 10) who met DSM-IV criteria for current MDD were enrolled in the clinical trial, with their treatment plan determined based on symptom severity: those with moderate depression symptom severity were recommended group PM+ alone; those with severe symptom severity were recommended a combination of group PM+ and antidepressant therapy (ADT).

Recipients of group PM+ were assigned to groups consisting of 2–8 adults and initiated on PM+ based on the location of the facility they attended. Each of the five sessions lasted 1.5–3.5 hours, following a manualized protocol developed by the World Health Organization [19]. Sessions included (1) Managing Stress, (2) Behavioral Activation, (3) Managing Problems, (4) Strengthening Social Support, and (5) Review of Techniques. Intervention components were communicated via stories, proverbs, group exercises, and homework assignments adapted to the local context. Participants were provided refreshments during each session but were not reimbursed for travel. Sessions were carried out by trained psychosocial counselors: this included counselors with bachelor’s level training as well as members of the local community with no prior experience (lay counselors), though participants were not told which counselors corresponded to which background. All counselors participated in a one-week comprehensive training on the manualized protocol and human subjects research, with refresher trainings held on a quarterly basis. To bolster protocol fidelity, clinical supervisors comprising mental health clinicians performed random supervision sessions, during which the supervisor would complete a PM+ fidelity checklist and meet with the counselor for review.

### Protocols

Participation in this study was based on purposive sampling. Participants were identified according to two criteria: first, participants needed to have received one or more group PM+ sessions as part of the clinical trial. Second, participants were required to be residents of Neno District as of 1 March 2023. All study participants were interviewed in Chichewa, the local language. We aimed to recruit participants until we achieved saturation – that is, the point at which qualitative analysis (described below) ceased to identify new themes or insights emerging from additional interviews.

To minimize social desirability bias in participant responses, interviews were conducted by a research team member who did not participate in any clinical aspects of the trial, including support or delivery of group PM+. The research team member, who held a Bachelor of Science in Public Health and was native Chichewa speaker, received comprehensive training to ensure proficiency with the semi-structured interview protocol. Four team members were involved in reviewing data on a weekly basis as data were collected – in order to provide routine feedback throughout the data collection process. The use of standard prompts and probes within the interview protocol helped maintain consistency for data collection and analysis.

The interview protocol covered four domains related to patients’ experiences and perceptions of PM+ as treatment for depression: understanding of depression as a mental health condition, experiences with depression treatment, assessment of PM+ effectiveness, and logistical considerations for attending group sessions and maintaining engagement over the course of treatment. By exploring these domains, the research team sought to gain a holistic understanding of the acceptability and feasibility of PM+, which the research team saw as requisites for PM+ to achieve its intended effect of reducing depression symptoms. Please see the Appendix for the full semi-structured interview protocol.

To ensure patient privacy and confidentiality during interviews, specific arrangements were made. For participants interviewed at health facilities, a dedicated unoccupied room was provided, while interviews conducted at patient homes were held in a quiet and private space. The interviews were captured on audio recorders to ensure accurate documentation, which were kept in a locked file cabinet accessible only by study team members. Interviews were manually transcribed and translated into English from Chichewa, relying on an expert bi-lingual translator with extensive prior experience. To ensure accuracy, back-translation from English to Chichewa was performed by another team member, and the team reviewed and compared both the original and back-translated Chichewa transcripts. The research team actively reviewed the transcripts as they became available and provided feedback to the interviewer to maintain data quality and consistency throughout the data collection process.

### Data analysis

In our analysis of the qualitative data from participant interviews, we conducted thematic content analysis using Dedoose software [29]. Three team members began by employing open coding to identify recurrent patterns, themes, and concepts within the dataset. This process involved systematically examining each interview transcript to generate an initial code set. Subsequently, the three team members applied axial coding to further refine the codes and establish connections between them, facilitating the development of overarching themes. Through an iterative process of coding and comparison, we identified several key themes that we believe captured the essence of participants’ experiences and perspectives.

### Ethical considerations

We obtained ethical approval from the Malawi National Health Science Research Committee (NHSRC) and the RAND Corporation Human Subjects Protections Committee in the US. All participants who agreed to participate in the study were provided with verbal informed consent and (when relevant) received travel reimbursement equivalent to 10 USD (12,000 MWK).

## Results

We enrolled 31 PM+ recipients as key informants, the point at which the study team determined we reached saturation of key themes. Among the consented key informants, 71% were female, and their ages ranged from 22 to 65 years (Table 1). The average PHQ-9 score at baseline was 13 and ranged from 10 to 22. The preponderance (94%) of key informants attended all five PM+ sessions, while remaining individuals (6%) attended 3–4 PM+ sessions. Half of participants (51%) received PM+ from a lay counselor rather than a professional counselor.

**Table 1.** Patient demographics characteristics.

Characteristic	Mean (SD)/Frequency (Percent)
**Sex**	
Female	22 (71%)
Male	9 (29%)
**Age**	
21–30	3 (10%)
31–45	16 (52%)
46–60	6 (19%)
>60	6 (19%)
**Depression Symptoms**	
PHQ-9 at Baseline	Mean = 13.4 (SD = 3.0)
**# PM+ Sessions Attended**	
3–4	2 (6%)
5	29 (94%)
**Counselor Type**	
Lay Counselor	16 (52%)
Professional Counselor	15 (48%)

**Figure 1.**
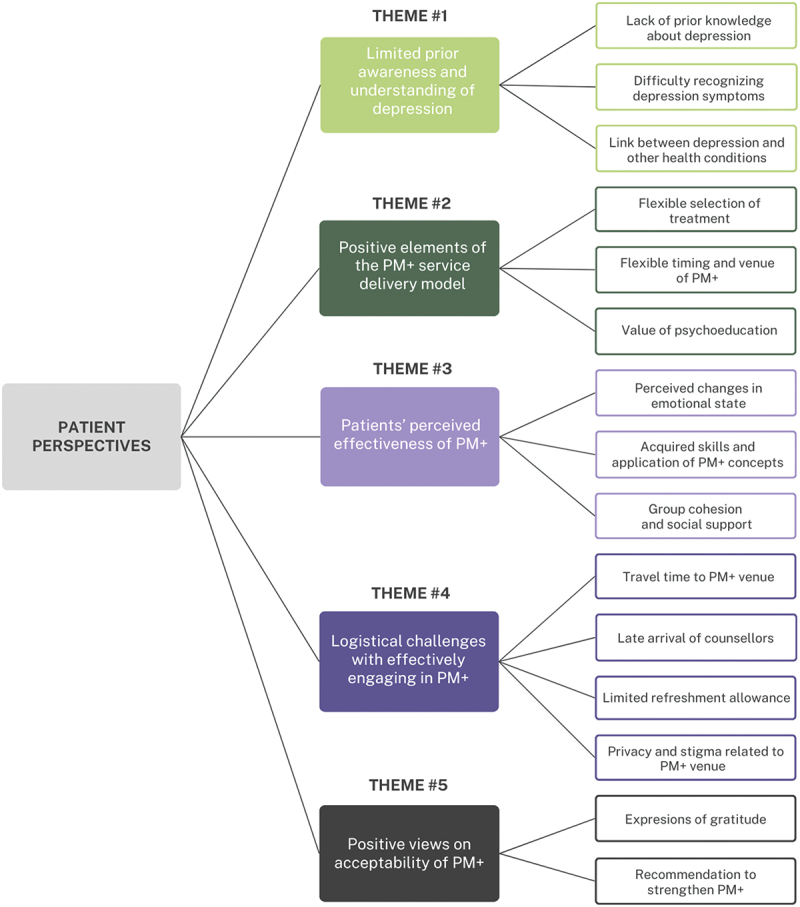
Diagram of qualitative themes and domains.

Following the thematic coding process described above, we identified five major themes: limited prior awareness and understanding of depression, positive elements of the PM+ service delivery model, patients’ perceived effectiveness of PM+, logistical challenges with effectively engaging PM+, and positive views on acceptability of PM+. These themes also covered various domains (or facets of the theme), which we describe in the following paragraphs as well as offering an overview in Figure 1.

### Theme 1: limited prior awareness and understanding of depression

A consistent theme that emerged across most interviews pertained to participants’ limited prior awareness and understanding of depression as a medical condition. In several instances, participants narrated that lack of knowledge about the causes, symptoms, and available therapies for depression was the reason they had never sought treatment. For example, a 44-year-old female participant remarked: ‘*For a year, I was just staying unaware of what was happening. I did not know whether it was a disease or not*.’ This lack of knowledge also contributed to difficulty recognizing depression symptoms, particularly physical symptoms.

A few participants recognized physical symptoms such as body pains, aches, and headaches, rather than emotional symptoms such as low mood and anhedonia. A 38-year-old male, shared:

A lot of times I had heart palpitations, headaches, and I would also have a lot of questions without answers because of what I was experiencing. Most times my blood pressure would be really high because of what I was thinking about as it was really hurting according to the circumstances I was facing. I would see that there is no future.

Others had internalized depression symptoms to such an extent that they considered it part of daily life. A 64-year-old female stated:

From way back, I have been experiencing these symptoms. I can’t really tell the period, as I just felt that was the way of my life so couldn’t tell how long I have experienced them. I would just say it was my life, when I feel a symptom, when I experience something depressing, that’s the way I was living.

The symptoms of depression that patients were experiencing were also associated with other chronic medical conditions for which they were taking medication. A few participants noticed depression symptoms only after diagnosis of a separate chronic condition, and they attributed depression symptoms to the chronic medical condition and associated medications A 42-year-old female participant reflected:

I have experienced these symptoms from last year. Because when I tested positive for HIV, I was usually depressed and sometimes with the way the marriage was going, so I was just staying inside. I stayed for a whole year before starting PM+.

### Theme 2: positive elements of the PM+ service delivery model

A second theme that emerged from the interviews was patients’ perceptions of the delivery model associated with PM+. Several patients expressed preferences for PM+ over antidepressant therapy (ADT), providing rationales that included: the group dynamic of therapy, aversion to the quick-fix nature of medications, the fear of pill burden of ADT, provider recommendations, and the influence of psychoeducation. For example, with respect to the group structure, one 44-year-old female participant remarked:

I saw that depression comes in because you are lonely. I saw that, maybe if I am in a group, it will be reduced. So, my choice was to meet with my fellow clients.

Regarding pill burden, one 64-year-old female offered:

I informed them that I am already receiving drugs for hypertension so I did not think receiving ADT was sitting well with me, but instead I opted for Group PM+ at that time.

A second dimension of the service delivery model that several clients perceived positively was flexibility during selection of the venue and time the PM+ groups would meet. For instance, a 65-year-old female participant stated:

The first day we met was the day we discussed about the time we would start our sessions; we were given the chance to choose the time we felt comfortable with.

Another 46-year-old female participant reflected:

We had the freedom to choose where we would be meeting. The counsellors listened to us to pick where we want and when we were picking the venue. We picked a place that we wouldn’t face any challenges. There weren’t any challenges, as the choice was in our hands.

At the outset of PM+, all individuals participated in psychoeducation that reviewed the objectives of group counselling, information on major depressive disorder, and the structure of the PM+ model. Several participants shared that psychoeducation steered them to PM+. For example, a 42-year-old male participant stated:

… They explained that we would be in groups for some days because we would meet for five times, on these five days, each day had its own topic… We were supposed to use it when we go home. It was really like a treatment indeed.

Ultimately, participants shared wide-ranging reasons for initiating PM+, concluding that provider engagement and psychoeducation played key roles in determining the treatment decision.

### Theme 3: patients’ perceived effectiveness of PM+

All key informants expressed that they perceived PM+ as effective. They reported enhancements in their lives through various dimensions, including alleviation of depressive and physical symptoms, diminished stress levels, and an increased ability to forge new relationships. Many participants shared the positive impact of the intervention on clinical symptoms and life outlook. For example, a 40-year-old male participant stated:

When I was diagnosed with depression, I thought it was the end of my life with the way my life was. But when I received PM+, I saw that I had found a new remedy. When I found this remedy, I saw that my life had started changing.

For several individuals, they identified a relationship between applying the skills and strategies of PM+ and positive outcomes. For instance, a 32-year-old female participant stated:

The other change that I saw is that, when we were taught on what to do once we are depressed, when I do it, I would feel calm… when someone talks to me in a way that would get me depressed, when I practice what they explained to us, I wouldn’t be depressed or be anxious. Everything would go well.

Several participants attributed the effectiveness of PM+ to group cohesion and social support, from counsellors and fellow participants. For example, a 29-year-old female participant shared:

[Counsellors] were good people. They would understand us. I felt good because they were free with us, and they were open with us so that we can open up with them

Reflecting more generally on this group dynamic, a 54-year-old male offered:

We would meet, and we were like a tea: there was unity, so it was pleasing that it would stop me from having bad thoughts. As I have said, I live alone, so when I come here, I would open up.

However, a few participants shared mixed feedback, including a 42-year-old male, who stated, *‘Of course as an individual you sometimes have that comfortability to freely explain to the doctor everything you are facing that in a group you cannot freely express. I saw that being in a group had its advantages and disadvantages.’* A fellow participant, a 32-year-old female, offered a slightly different expression:

The advantages of being in an individual format is that you can be able to open up about how you really feel … But being in a group you are able to hear your fellow client’s problems and see that yours are better off, as I have explained.

### Theme 4: logistical challenges effectively engaging with PM+

All participants shared indicated logistical challenges associated with PM+. These included: travel distance to PM+ venues, the late arrival of the counsellors, limited allocated allowance, issues regarding privacy and stigma that could derive from lack of privacy. Concerning travel distance, most participants reported traveling far to the group PM+ venue. For example, a 40-year-old female reported:

The distance was quite long, and we even had to cross a bridge to get there. Despite this, because we were really eager for counseling, we did not perceive it as too far.

This frustration was compounded by counselors who would periodically arrive late to sessions, noted by a several interviewees. For instance, a 47-year-old female participant narrated:

What I did not like is that, when we arrive there, maybe when we have been given a time like let’s say 8am, the counsellors would arrive late, maybe around 10am.

Another salient challenge pertained to the role of money. Participants were given stipends for refreshments; yet, when coupled with long distances that individuals travelled, a few participants shared that they felt like the stipend amount was inadequate. One 65-year-old female reflected:

What was difficult was the money we got. They gave us money to buy food, but we wouldn’t buy the food because when coming we were walking so we thought it is better we rest when returning home. We should ride a motorbike.

Group PM+ also posed a challenge to some participants due to privacy concerns. Although several participants shared a positive disposition on PM+ venues, a few were concerned. One participant perceived that the venue used for PM+ would even contribute to stigmatisation of people with mental health problems. A 30-year-old female participant reflected:

The other thing that was disappointing, something that was a bit challenging, was the venue because when the time arrived at the very beginning of the sessions, people would say that the organization is dealing with mentally disturbed people whose mind is not functioning. When people from there saw me, the whole hospital said I was a confused person, and I am now in the group of people that are mentally disturbed.

### Theme 5: positive views on acceptability of PM+

Overall, all participants expressed acceptance of PM+ as a treatment option. In most instances, this came as an expression of gratitude and recommendations of treatment to others. A 40-year-old female stated:

I can recommend to them because I have received PM+ before and I know everything about it. Even now, I assist my relations on the things I learnt. I am able to assist them since I received PM+. I advise them on what to do for them not to be depressed and indeed it helps in my life.

Acceptability was also conveyed, positively and (to a lesser extent) negatively through recommendations to strengthen and expand PM+. For example, several participants viewed poverty as a major factor driving their depression and wanted PM+ to accommodate this. A 65-year-old female participant shared: *'PM+ is good but we wanted assistance in terms of money.'* Another individual, a 54-year-old male, shared: *'If there was something to do, maybe having money for business or to buy fertilizer, maybe we wouldn’t be depressed and we would also be assisted.'*

Others shared a strong appreciation for the routine meet-ups with other individuals and wanted to expand this through continued interactions with counsellors. A 54-year-old male participant stated: ‘*I feel like we should be meeting frequently or meeting so that we should discuss on what we need, which can help us in our life.’* This statement reflected the positive impact of the relationships among each participant cohort.

## Discussion

This study investigated the views of patients with MDD who received group PM+. The findings identified several key themes that emerged from patients’ perspectives, including their lack of understanding about MDD, patients’ assessment of the PM+ model, perceived efficacy of PM+, logistical challenges encountered with PM+, and overall acceptance and endorsement of PM+.

Regarding prior awareness and understanding of depression, patients generally exhibited limited mental health literacy prior to PM+, a prevalent issue in Malawi. This finding aligns with previous research conducted in Lilongwe, Malawi, where authors found that knowledge about the symptoms, causes, and treatments of depression varied widely among both mental health providers and patients [30]. Similarly, researchers have noted that – in many low- and middle-income countries – public knowledge of mental health conditions tends to be generally poor or incomplete [31]. Insufficient knowledge regarding the causes, symptoms, and available therapies for depression is likely to hinder the utilization of mental health services, even when they are accessible. This may also negatively affect scaling up of PM+ as an intervention [32]. It is therefore imperative that psychoeducation should be provided to patients who are screened and (separately) diagnosed with MDD. Psychoeducation may also improve patient adherence to treatment, including session attendance, by generating greater intrinsic motivation among participants [33]. In a similar vein, it is well understood in the research literature that depression can manifest as physical symptoms such as general body pains, aches, and headaches [34]. Our study elucidated that individuals with limited knowledge about depression who suffer from MDD may easily misconstrue these manifestations as stemming from other physical ailments. This underscores the necessity of improving awareness and education about the multifaceted presentations of depression, particularly in psychosomatic forms, to enable more accurate identification and timely intervention.

Given that PM+ is a novel intervention in Malawi, patients were provided with psychoeducation about the intervention on the same day they received their depression diagnosis. The non-aversive nature of PM+, together with its absence of side effects and the avoidance of additional medication burden, likely contributed to its preference over pharmacological treatments. This finding is especially relevant in the specific context of this study – as patients were concurrently managing other chronic medical conditions, requiring a regimen of multiple medications and treatment protocols. The provision of psychoeducation helped patients understand the delivery method and anticipate logistics associated with receipt of the intervention.

Efficacy of PM+, in terms of clinical symptom reduction, has been positively concluded by studies across diverse settings. For instance, studies conducted in Nepal, Philippines, sub-Saharan Africa, and Eastern Europe have all reported significant reductions in symptoms of depression and anxiety, along with improvements in daily functioning [18,35,36]. Similarly, a study done in the Netherlands found that PM+ was generally received positively by stakeholders involved in a clinical trial for adult Syrian refugees [37]. These prior studies, as well as the testimony of the participants in the present study, outline several suggestive mechanisms by which enhanced emotional wellbeing may be achieved. This includes the emphasis of PM+ on concrete behavioral strategies in response to ongoing problems [38], as well as the development of a broader repertoire of coping mechanisms in the face of adversity. Future studies might test these theories directly through the application of additional survey instruments and structural equation modeling.

In the present study, participants also shared that the group format contributed to the perceived effectiveness of PM+. This may be attributable to the fact that the group format helped normalize mental health experiences and reduced mental health-related stigma [20], and it promoted mutual validation of individual experiences [39]. Furthermore, participants were able to identify a causal mechanism leading to this improvement process: namely, that they were able to recall and apply strategies learned in the context of group PM+ sessions. This provides suggestive testimony that the tools embedded in PM+ had practical applications. Participants also noted that flexibility in terms of choosing venues and the timing of meetings ensured broad attendance for all group members.

Despite these stated successes of PM+, there were evident challenges highlighted by participants, including physical challenges navigating the rough terrain in Neno District to travel to sessions, as well as clinical challenges such as limited venue spaces and corresponding lack of privacy. Previous studies have singled out transportation challenges as a barrier to the successful implementation of PM+ [40]. One potential response to this would be the development of internet- or phone-based participation for those who are unable to attend in-person for specific sessions. Prior literature indicates that virtual participation in mental health interventions corresponds to depression symptom reduction and improved social functioning [41,42]. In the present study, participants also stated that counsellors arrived late for sessions, often a product of challenges with transportation and weather. A potential solution here may be to develop multiple modes of transportation for counselors in the event the primary mode fails, and to communicate in real-time with participants on expected times of arrival. Conversely, participants noted that the relationships with their provider and peers were a core aspect of the intervention, and many respondents advised integrating follow-up care systems in tandem with group psychotherapy. These challenges and proposed revisions reflect common barriers to care in community-based mental health care settings, and they elucidate areas for improvement for future iterations of PM+ in Malawi.

Overall, the perceived acceptability and feasibility of PM+ is highly encouraging. The participants’ willingness to recommend PM+ to others stands out as one as a key achievement, as it shows the potential expansion and sustainability of PM+ in this low-resource setting. Buy-in from beneficiaries of the intervention offers an opportunity for leveraging this to expand access to psychotherapy among others within the community. As demonstrated in other studies, stigma reduction may be a mechanism by which participants develop a willingness to advocate for treatment among peers and family members [43]. Across diverse settings, including Malawi, PM+ has demonstrated effectiveness and garnered positive reception when delivered by various cadres [4,12]. As in the present study, a prior trial conducted in Nepal employed lay counselors for group PM+ and found meaningful improvements among recipients [18]. In Kenya, PM+ sessions were conducted via phone [44], showcasing the potential adaptability of the intervention to promote continuity of care in remote villages such as those in Neno District, Malawi.

### Limitations

We acknowledge certain limitations that should be considered when interpreting the findings of this study. First, data collection relied solely on individual interviews, and the absence of focus group discussions may have limited the depth of shared perspectives and experiences. Additionally, some patients were interviewed one year after participating in the PM+ session, potentially leading to recall bias, as they may have forgotten less salient aspects of their experience. Lastly, interviews were conducted in Chichewa (the local language) and translated to English. As such, some of the nuance in perspectives that were offered by participants may have been lost in translation.

### Conclusion

In summary, this study finds that a locally adapted version of group PM+ for treating MDD in adult patients with chronic medical conditions is highly suitable in rural Malawi, according to the study participants. The findings underscore the promise of task-shifting and care integration as strategies to improve mental health outcomes in low-resource sub-Saharan African settings. Task shifting of PM+ to local counselors not only promoted economic feasibility but was also widely accepted by participants and viewed as an asset for fostering social cohesion. The group format proved beneficial for reducing stigma and normalizing experiences of depression and may therefore be a relevant strategy in other collectivist settings.

Participants also expressed a need for follow-up care and further linkages to social and financial support, highlighting the potential for future research to embed mental health services in broader service delivery models that address social determinants of health. Looking ahead, researchers and program planners might also investigate longer-term outcomes of PM+ to assess whether symptom reductions and functional improvements are sustained over time, as well as how to tailor PM+ to local constraints such as participants facing poverty and limited transportation, including the deployment of hybrid virtual and in-person delivery to bolster continuity of care.

## Appendix

### Appendix 1 Semi-Structured Key Informant Interview Protocol Questions

#### Background Information

Thank you for agreeing to participate today – we are hoping your feedback will help us to strengthen PM+ services in the future. To begin with, we would like to ask you a bit about your life experiences with depression.

1. Could you share your experiences being diagnosed with clinical depression and your depression symptoms?

2. When did you first receive a diagnosis of clinical depression?

3. How long did you experience these symptoms prior to starting PM+?

4. What previous treatment/services did you receive for depression before enrolment in PM+ and/or ADT?

5. How were you informed/who talked with you about the intervention?

6. What were you told about group PM+ before starting the intervention? What did these individuals say?

a. Probe: informed about the starting group PM+

b. Probe: organized into a group session and the location of group PM+

#### Treatment Modality

I would like to talk to you about your opinions and feelings related to treatment. There are no right or wrong answers. Your feedback will be very helpful so that we can improve our care. Can you please share your feedback on:

7. The group format of the intervention, rather than one-on-one with a clinician?

8. Your feelings about privacy and confidentiality of sessions?

9. The duration and frequency of sessions?

10. What did you like most/least about the format?

#### PM+ Logistics

11. What is your feedback on the logistics of the intervention? Specifically, I’d like your reflections on:

12. Your feelings about the PM+ therapy venue: what did you like/dislike, if anything?

13. Travel to the venue: was this easy/hard? Why?

14. Payment compensation for food: did this help/was this sufficient?

#### Treatment Contents/Recommendations

15. What were your feelings about the PM+ sessions: What did you like? What did you dislike?

a. Probe: Can you give examples?

16. What would you suggest that we change about PM+ to make it better, if anything?

17. If it happens that you are diagnosed with depression again in the future, would you choose PM+ as your desired treatment? If YES, why? If NO, why?

18. Would you recommend PM+ treatment for depression to a family member or friend? How come?

19. Do you have anything you would like to share with us concerning PM+ as a mode of treatment for depression?

#### Conclusions

Great, thank you for your time today!

20. Before we end, do you have any other reflections about your experiences with PM+ that you would like to share with us?
